# Hypoxemia prediction model based on XGBoost during sedation for gastrointestinal endoscopy

**DOI:** 10.3389/fmed.2025.1714512

**Published:** 2026-01-12

**Authors:** Rong Zhao, Zheng Chen, Qingyu Teng, Tao Xu, Qi Li, Helin Gong, Hongjun Ji, Hui Zhang

**Affiliations:** 1 Department of Anesthesiology, Shanghai Sixth People's Hospital Affiliated to Shanghai Jiao Tong University School of Medicine, Shanghai, China; 2 Shanghai Jiao Tong University Paris Elite Institute of Technology, Shanghai Jiao Tong University, Shanghai, China

**Keywords:** endoscopy, hypoxemia, machine learning, sedation, XGBoost

## Abstract

**Introduction:**

Hypoxemia is the most common complication of sedated gastrointestinal endoscopy and can lead to serious consequences. Predicting and preventing hypoxemia remains challenging. Accurate prediction using integrated clinical data and artificial intelligence shows great potential. This study aimed to develop a robust, interpretable, and generalizable Machine Learning (ML) model with acceptable performance for predicting hypoxemia during sedated gastrointestinal endoscopy.

**Methods:**

This prospective study included 647 adult patients who underwent sedated gastrointestinal endoscopy at Shanghai Sixth People's Hospital, affiliated with Shanghai Jiao Tong University School of Medicine, between January and May 2025. We employed a combination of statistical and ML techniques, including Pearson correlation analysis, *T*-test, Chi-square test, Levene test, SHapley Additive exPlanations (SHAP) values, and eXtreme Gradient Boosting (XGBoost) feature importance metrics, for feature selection. Prediction models were developed using XGBoost algorithms, and its performance was evaluated using Accuracy, Precision, Recall, F1-score, and Receiver Operating Characteristic Area Under the Curve (ROC–AUC). After identifying the optimal model, a hypoxemia prediction model was established and validated. We also analyzed the performance of combined features to create innovative features.

**Results:**

The XGBoost model demonstrated the best performance, achieving an accuracy, recall, and F1-score of 0.91 and an ROC–AUC of 0.74 using the selected features. Feature importance analysis identified 29 key features, including 26 traditional features and three innovative features introduced in this study, where Body Mass Index (BMI), waist circumference, neck circumference, age, baseline SpO_2_ contribute most significantly. Model performance improved when applied to a more balanced dataset of 647 samples, underscoring the importance of sample size in model accuracy.

**Conclusion:**

We present a robust XGBoost-based hypoxemia prediction model that can help clinicians identify at-risk patients during sedated gastrointestinal endoscopy. The model's performance highlights the potential of artificial intelligence to enhance patient safety and clinical decision-making. Future studies should focus on refining the model using larger and more diverse datasets to improve predictive accuracy and clinical applicability. Additionally, methods such as latent-space analysis will be explored to address class imbalance.

## 1 Introduction

Gastrointestinal endoscopy is a cornerstone in the diagnosis and treatment of many gastrointestinal disorders and is considered the gold standard for early detection of gastric and colorectal cancers (1). Sedated endoscopy facilitates smoother procedures for endoscopists, reduces technical challenges, and improves the detection of subtle lesions, thereby meeting a wide range of clinical needs (2, 3). However, with the increasing use of sedation in gastrointestinal endoscopy, it is important to recognize that sedation carries a considerable risk of complications (3–5), with hypoxemia being the most significant adverse event (6). Reported incidence rates of hypoxemia during sedated gastrointestinal endoscopy vary widely, ranging from 1.8 to 70.0% (7, 8). Hypoxemia is a serious complication that poses significant risks to patients, including cardiac arrhythmias, cardiac arrest, and cerebral ischemia (9–12). Accurate risk assessment of hypoxemia is therefore essential to support clinicians in resource allocation, planning, and execution of intervention strategies, and preparation for emergencies. Moreover, it plays a critical role in reducing the incidence of severe hypoxemia and improving overall patient outcomes (13).

In a preliminary study, three regression analysis models—logistic regression, linear discriminant analysis, and fisher discriminant analysis—were applied to 394 samples. The average predictive performance for positive samples was accuracy = 0.50, precision = 0.97, recall = 0.17, and F1-score = 0.29. These findings demonstrated that traditional regression analysis performed poorly in predicting samples, despite good performance in predicting negative samples.

The application of Artificial Intelligence (AI), including ML and natural language processing, in clinical disease prediction has revolutionized healthcare by improving diagnostic accuracy, treatment planning, and personalized care (14, 15). ML-based prediction models can analyze critical clinical factors and integrate prognostic features. ML techniques specifically to predict hypoxemia during sedated gastrointestinal endoscopy remain scarce (16). Therefore, a robust and interpretable ML-based hypoxemia prediction model is needed for patients undergoing sedated gastrointestinal endoscopy. In this study, we designed prediction models using Random Forest (RF), Self-paced Ensemble Classifier (SPE), and XGBoost algorithms. Compared to traditional regression analysis, ML-based models offer distinct advantages.

The objective of this study was to develop a simple, robust, and generalizable ML model with acceptable performance for predicting hypoxemia during sedated gastrointestinal endoscopy. This model aims to assist clinicians in prospectively identifying hypoxemia risk, stratifying patient assessments, and implementing timely interventions to mitigate complications.

## 2 Materials and methods

The study protocol complied with the principles of the Declaration of Helsinki. This study was approved by the ethics committee and review board of Shanghai Sixth People's Hospital of Shanghai Jiao Tong University School of Medicine. Written informed consent was obtained from all participants. Chinese Clinical Trial Registry Identifier: ChiCTR2400094393. This prospective cohort study included patients who underwent sedated gastrointestinal endoscopy (gastroscopy, colonoscopy, or both) at Shanghai Sixth People's Hospital between January and May 2025. Patients were included according to the following criteria: ≥18 years old; American Society of Anesthesiologists (ASA) class I–III. Exclusion criteria were as follows: pregnancy, severe cardiopulmonary diseases (including acute myocardial infarction, severe aortic stenosis, acute exacerbations of chronic obstructive pulmonary disease, etc.), presence of acute upper respiratory tract infection, and unavailability of complete data.

### 2.1 Procedures

All patients fasted for at least 8 h before gastrointestinal endoscopy and remained in the lateral position throughout the procedure. Each patient received 3 L/min oxygen via nasal cannula for 2 min before sedation and underwent cautious monitoring of pulse oximeter (SpO_2_), blood pressure, and electrocardiography during the procedure. Anesthesiologists used propofol (1–2 mg/kg) combined with fentanyl (50–100 μg) for induction, with additional propofol administered to maintain stable sedation depth. If oxygen saturation dropped below 95% during gastrointestinal endoscopy, proper attachment of the finger probe was first verified to rule out equipment errors. To ensure patient safety, interventions were performed as follows: increasing flow to 6–8 L/min, opening the airway with a jaw-thrust maneuver, providing mask ventilation, and performing tracheal intubation if hypoxemia could not be corrected. During the procedure, appropriate interventions were administered based on the patient's vital signs, including atropine for heart rate augmentation and ephedrine for blood pressure support, etc.

### 2.2 Endpoints and data source

The primary endpoint was SpO_2_ ≤ 95%. According to the World Society of Intravenous Anesthesia (World SIVA) criteria (17), SpO_2_ between 90 and 95% during sedated gastrointestinal endoscopy indicates subclinical respiratory depression. Therefore, in this study, SpO_2_ ≤ 95% can be used as an early warning signal of hypoxia to alert anesthesiologists to intervene. Hypoxemia was defined as SpO_2_ < 90% (17). Data were obtained from the hospital's clinical information system and included prospectively collected variables on patient characteristics, anesthesia protocols, and endoscopic procedures. In this study, 394 samples, with 42 (10.66%) positive samples and 352 (89.34%) negative samples, have been collected to conduct feature statistical analysis, feature selection and model construction. To validate the generalization of model and the benefit of XGBoost, 253 samples are added into the original 394 samples, forming totally 647 samples, with 72 (11.13%) positive samples and 575 (88.87%) negative samples.

### 2.3 Study design

**Figure 1:**
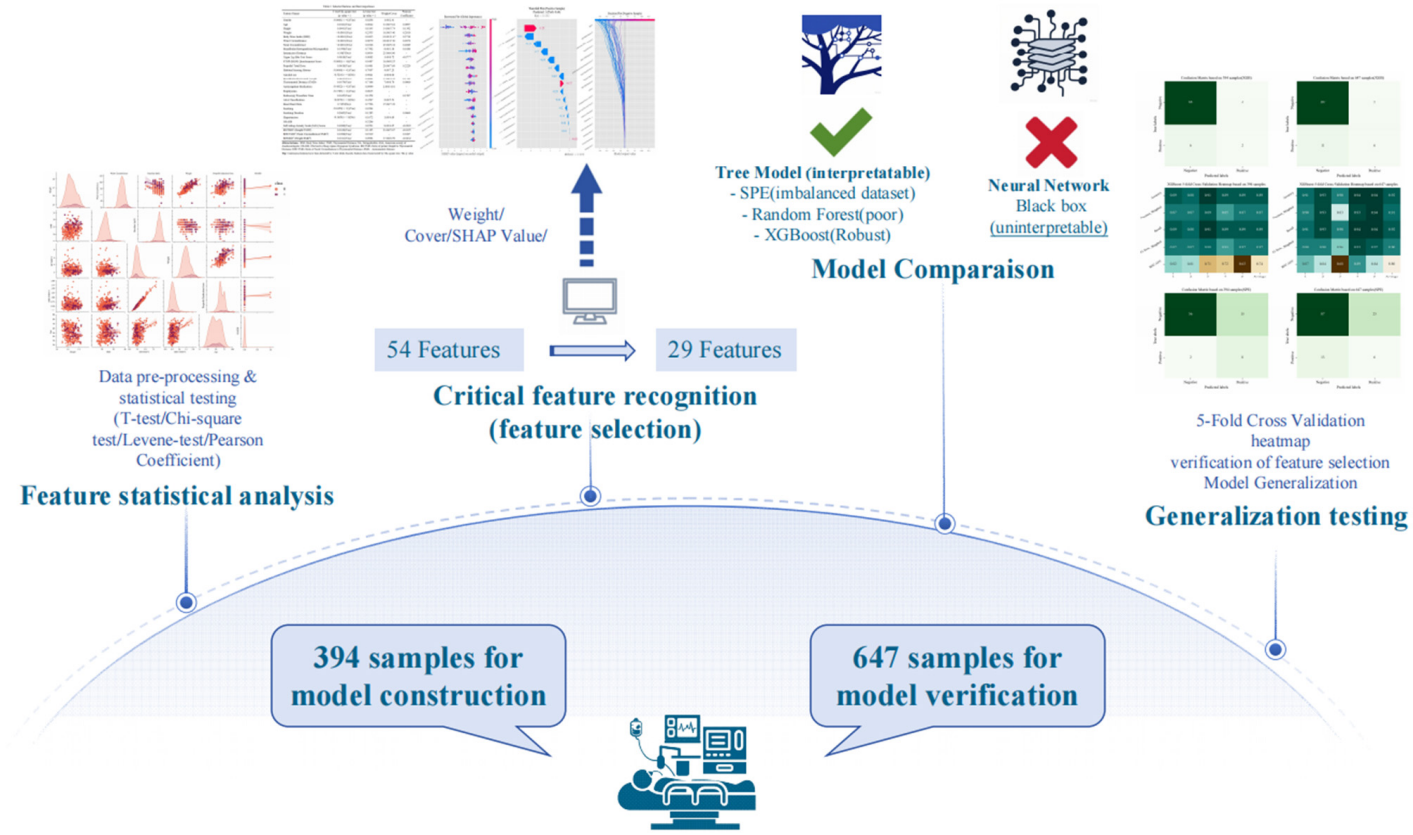
Schematic procedure of modeling hypoxemia prediction during sedation for gastrointestinal endoscopy.

This study aimed to determine whether patients undergoing sedated gastrointestinal endoscopy were at risk of intraoperative hypoxemia, framed as a binary classification problem. By analyzing key contributing factors and applying predictive modeling, the system sought to provide an accurate risk assessment. This ML approach was intended to support clinicians in evaluating risk before endoscopy, thereby enhancing patient safety and optimizing outcomes. The study process can be summarized in four steps: feature statistical analysis, critical feature recognition, model comparison, generalization testing (Figure 1).

### 2.4 Feature statistical analysis

Statistical analysis and model training are depend on a high-quality dataset, which requires the data preprocessing first. The data preprocessing encompasses four processes: categorical encoding, missing-value columns dropping, data normalization and training-test set split. In the categorical encoding stage, binary features (e.g., sex) were encoded as 0 and 1, whereas categorical variables with more than two levels (e.g., ASA classification) were assigned consecutive integers starting at 0 and ending at the total number of categories minus one. Label encoding is applied across all ML models, including XGBoost and logistic regression mentioned in the introduction. In view of the critical prognostic value of extreme categories, all such categories—no matter how infrequent—were deliberately retained. In the missing-value stage, the features with more than 30% missing samples have been dropped, while the numerical missing values in the other columns have been filled by K-Nearest Neighbor (KNN) and the categorical missing values are imputed by mode. The missing-value filling is conducted before the training-test set split.

After the data preprocessing, *T*-test, Chi-square test, Levene test and Pearson's correlation are used to conduct the statistical analysis of features for the purpose of the preliminary analysis of feature importance. *T*-test exhibits a well testing efficience in the dataset with a moderate sample size, which is suitable for this study. Chi-square test is a cornerstone non-parametric statistical procedure widely used in research to determine whether a significant association exists between two categorical variables, which is more interpretable. In view of the fact that the method which is easy to explain is needed in the clinical application, Chi-square test is used. In this study, the significance level is set as 0.05, which is widely used in other studies. If *p*-value of a feature is inferior to the significance level, we can reckon this feature having significant difference between positive and negative samples. Levene test is preferred for its robustness to departures from normality, therefore it is used to analyse the features with anormal distribution. Pearson's correlation is used to quantification the relationship between features and the SpO_2_, therefore concluding the feature importance.

### 2.5 Critical feature recognition (feature selection)

In this section, XGBoost (18), which is advanced tree model, is used to recognize the critical features, achieving the reduction of dimensionality of features.

#### 2.5.1 Why we choose XGBoost

In recent years, advances in computer science have established ML as a powerful alternative to traditional mathematical modeling, because of its exceptional learning capabilities (19, 20). Among numerous ML algorithms, tree model is spreadly used in the clinical issues in view of its rapid convergence in the small-size dataset and the well interpretability, especially it exhibits a prominent capacity to deal with the nonlinear relationship (21). Among the tree models, XGBoost is a state-of-the-art model which can detect the best performance of tree model without dealing with imbalance. The dataset employed in this study is characterized by a limited sample size and pronounced class imbalance-precisely the conditions under which XGBoost demonstrates its principal strengths, and thus constitutes the rationale for its selection.

#### 2.5.2 Feature contribution

After the statistical analysis of features, the features with significant difference between groups are further tested by the method of ML. XGBoost is an advanced ML model that can effectively highlight the importance of features using two metrics: weight and cover. Weight represents the times that a feature used in the branch split, while the cover calculates the sum of the covers of the leaf node generated when a feature is used for splitting in all trees.

In this step, XGBoost is trained under the default hyper-parameters, with 100 base estimators. After training the model, SHAP, which is an index designed to interpret the tree model, is used to recognize the contribution of features again. In the SHAP analysis, beeswarm, waterfall and decision figure have been plotted to observe the decision process. The bandwidth in the beeswarm represents the contribution of one feature and the influence of value can be concluded through the color. Waterfall is always used to interpret a sample, where the positive and negative contribution of features are shown by the arrows. Decision figure illustrates the process of branch split in the training. Typically, waterfall was plotted by a distinctive positive sample while decision figure was plotted by negative samples.

#### 2.5.3 Justification of selected features

To validate the rationale of the feature selection, all selected features, stratified features and the features with synergy were respectively input into the XGBoost with the default parameters, in comparison with all features without selection.

Feature stratification. Based on clinical experience and literature review, our study stratifies selected features into “very important factors” (proven to be independent risk factors), “important factors” (already proven as risk factors in some studies) and “potential risk factors.” In each group, features will be first tested distinctively, followed by the combination testing.

Feature synergy. Through clinical experience, clinicians observe that certain combinations of specific indicators can enhance the accuracy of hypoxemia prediction. Based on this insight, our study systematically explores feature combinations across the entire feature set, selecting those combinations that yield the best results.

- Firstly, features with varying levels of importance were input into the model using the default hyper-parameters.
- Secondly, we incorporated innovative airway features to predict hypoxemia and assess their contribution to the model.
- Thirdly, we experimented with incorporating various feature combinations into the model using the default hyper-parameters.

### 2.6 Model comparison

In view of the benefit of tree model for this study (such as the well interpretability and the simplicity), two mainstream tree models will be used to compare with the XGBoost. RF, as a ML model built on the foundation of decision trees, can reduce the imprecision that arises when relying on a single decision tree. SPE (22), which aims at the dealing of imbalanced dataset, is selected to deal with the imbalanced dataset.

To quantify the model performance, five indicators are used, which are respectively Accuracy, Precision, Recall, F1-score, and ROC-AUC. All the indicators are used weighted version, that is to say that we take the unbalanced labels into account. In clinical practice, a high False Positive Rate (FPR) leads to the consequence that more patients will be misdiagnosed as at risk, resulting in additional precautionary medical treatments. While this may impose extra effort, it does not pose a significant threat to patient health. Conversely, a high False Negative Rate (FNR) is more critical, as patients misdiagnosed as low risk may not receive necessary interventions, increasing their vulnerability to complications during gastrointestinal endoscopy. So there is less tolerance for false negative (23). To achieve this, the model should prioritize high Recall and F1-score while maintaining strong performance across other metrics. Additionally, ROC-AUC serves as a complementary measure to evaluate the overall capacity of the model.

In the period of model comparison, models have been trained to the best performance through hyper-parameter adjustment. The common hyper-parameters of tree model include n-estimator, max-depth, minimum weight of leave, subsample number, learning rate (α), γ, λ. Five-fold cross-validation (CV) and Grid Search (24) are used to tune the hyper- parameter of ML models so as to enhance their performance. In each iteration of CV, 20% of the data is used as the test set, while the remaining 80% is used for training. The verification and performance of models below are both based on the best-performance hyper-parameters.

### 2.7 Generalization testing

Considering Random Forest and XGBoost perform poorly in dealing with the imbalanced data set, enlarging the sample size to validate the model performance is necessary. To achieve this goal and verify the generalization of trained model, the confusion matrix will be drawn based on 394 samples and 647 samples, of which 647 samples contain more positive sample that can alleviate the imbalance impact.

Constructing a model using a fixed training set and test set can lead to overfitting. To address this issue, it is essential to use different training and test sets. The five-fold CV method helps mitigate overfitting by splitting the dataset into five subsets. Besides, model's generalizability to imbalance will be obtained by comparing the result of five-fold CV based on dataset with different sample number. Taking the differentiated emphasis between XGBoost and SPE into account, a comparative test will be conducted on the two models and five-fold CV will be carried out. This comparative test will contribute to the future enhancement of models. In order to visualize the generalization test, heatmap, which can clearly show the distributional difference of each fold, will be utilized in our work. If heatmap indicates a balanced distribution among the five folds, trained model possesses a relatively strong generalizability.

## 3 Statistical analysis

All analyses in this study were performed using Python 3.13.1 and Excel. Excel was used for data preprocessing. Scipy.stats.pearsonr in Python was used to calculate Pearson's coefficient during scikit-learn. Model_selection.cross_val_score was used to calculate the CV results. Scikit-learn was used to construct a RF model and generate a classification report. The XGBoost Classifier was used to construct XGBoost. A self-paced ensemble classifier was used to construct the SPE. SHAP values were applied to interpret tree models. Matplotlib and Seaborn were used to plot figures.

## 4 Results

### 4.1 Feature importance and selection

Statistical analysis provided an initial gauge of feature importance: hypothesis testing discerned whether a feature differed significantly between the two groups, whereas Pearson's correlation coefficient subsequently quantified the strength of the linear association between each continuous feature and the prediction target. Based on the results of ML identification, 29 key features were finally identified. Among these, 26 were traditional features and three were innovative features introduced in this study. According to the importance, these 29 features are stratified to three classes, where BMI, waist circumference, neck circumference, age, baseline SpO_2_ are the most important five features.

- Very important risk factors: BMI, waist circumference, neck circumference, age, baseline SpO_2_, Thyromental Distance (TMD), Sternomental Distance (SMD), habitual snoring history.
- Important risk factors: Obstructive Sleep Apnea Hypopnea Syndrome (OSAHS), smoking history, Self-rating Anxiety Scale (SAS) score, basal heart rate, propofol total dose, hypertension, Height/TMD^2^ (RH/TMD^2^), Height/SMD^2^ (RH/SMD^2^), endoscopist level, ASA classification, mandibular retroganthism/micrognathia.
- Potential risk factors: Other features.

The 29 features are listed in Table 1. From this table, BMI, waist circumference, and neck circumference ranked in the top three for weight and coverage (75.21, 71.03, and 61.77%, respectively). In addition, BMI showed a high Pearson's coefficient and demonstrated significant correlations across the three tests. Besides BMI, neck circumference, and waist circumference also showed significant differences (*p* < 0.0001) between the positive and negative groups in the *T*-test and Chi-square test. In addition, waist circumference had the highest Pearson's coefficient, indicating its strong contribution to hypoxemia prediction. Our innovative features, which are RH/TMD^2^ and RH/SMD^2^, also count numerously (about 60 times) in terms of the cover obtained by ML algorithm, showcasing a great potential in the hypoxemia prediction.

**Table 1:** Selected features and their importance (sample = 394).

**Feature names**	***T*-test/chi-square test (*p*-value = )**	**Levene test (*p*-value = )**	**Weight/cover**	**Pearson coefficient**
Gender	/0.0001 (α = 1; yes)	0.0258	0.11/35.21	–
Age	0.0321 (yes)/	0.0642	0.07/55.20	0.0097
Height	0.0041 (yes)/	0.8145	0.09/40.59	0.1392
Weight	< 0.0001 (yes)/	0.2555	0.12/53.38	0.2810
BMI	< 0.0001 (yes)/	0.0435	0.17/75.21	0.2718
Waist circumference	< 0.0001 (yes)/	0.0079	0.15/71.03	0.2870
Neck circumference	< 0.0001 (yes)/	0.0348	0.14/61.77	0.2689
Mandibular retrognathism/micrognathia	0.3598 (yes)/	0.7592	0.07/34.96	0.1686
Interincisor distance	0.5687 (no)/	0.0934	0.08/48.83	–
Upper lip bite test score	0.0818 (yes)/	0.0082	0.08/21.64	−0.0777
STOP-BANG questionnaire score	/0.0003 (α = 6; yes)	0.0487	0.09/42.66	–
Propofol total dose	0.0018 (yes)/	0.0401	0.09/46.66	0.2229
Habitual snoring history	/0.0048 (α = 1; yes)	0.7007	0.11/34.36	–
Alcohol use	/0.5216 (α = 1; no)	0.9621	0.06/24.01	–
Mandibular horizontal length	0.0017 (yes)/	0.6964	0.11/47.10	0.1142
TMD	0.0179 (yes)/	0.7106	0.07/23.86	0.0860
Anticoagulant medication	/0.4812 (α = 1; yes)	0.0999	0.14/14.00	–
Emphysema	/0.3785 (α = 1; yes)	0.0015	–	–
Endoscopy procedure time	0.0345 (yes)/	0.1258	0.05/17.23	0.1507
ASA classification	/0.6458 (α = 1; no)	0.4567	0.08/12.62	–
Basal heart rate	0.7652 (no)/	0.7526	0.07/15.02	–
Smoking	/0.0459 (α = 1; yes)	0.0504	0.05/14.97	–
Smoking duration	0.2685 (yes)/	0.1285	0.09/27.76	0.0860
Hypertension	/0.1628 (α = 1; no)	0.1072	0.09/25.47	–
OSAHS	–	0.5206	–	–
SAS score	0.4888 (yes)/	0.8523	0.05/22.58	−0.0265
RH/TMD^2^	0.0140 (yes)/	0.1395	0.09/52.25	−0.0395
RHC/TMD^2^ (neck circumference/TMD^2^)	0.1698 (yes)/	0.1919	0.05/14.81	0.0367
RH/SMD^2^	0.1811 (yes)/	0.0994	0.08/63.33	−0.0232

**Figure 2:**
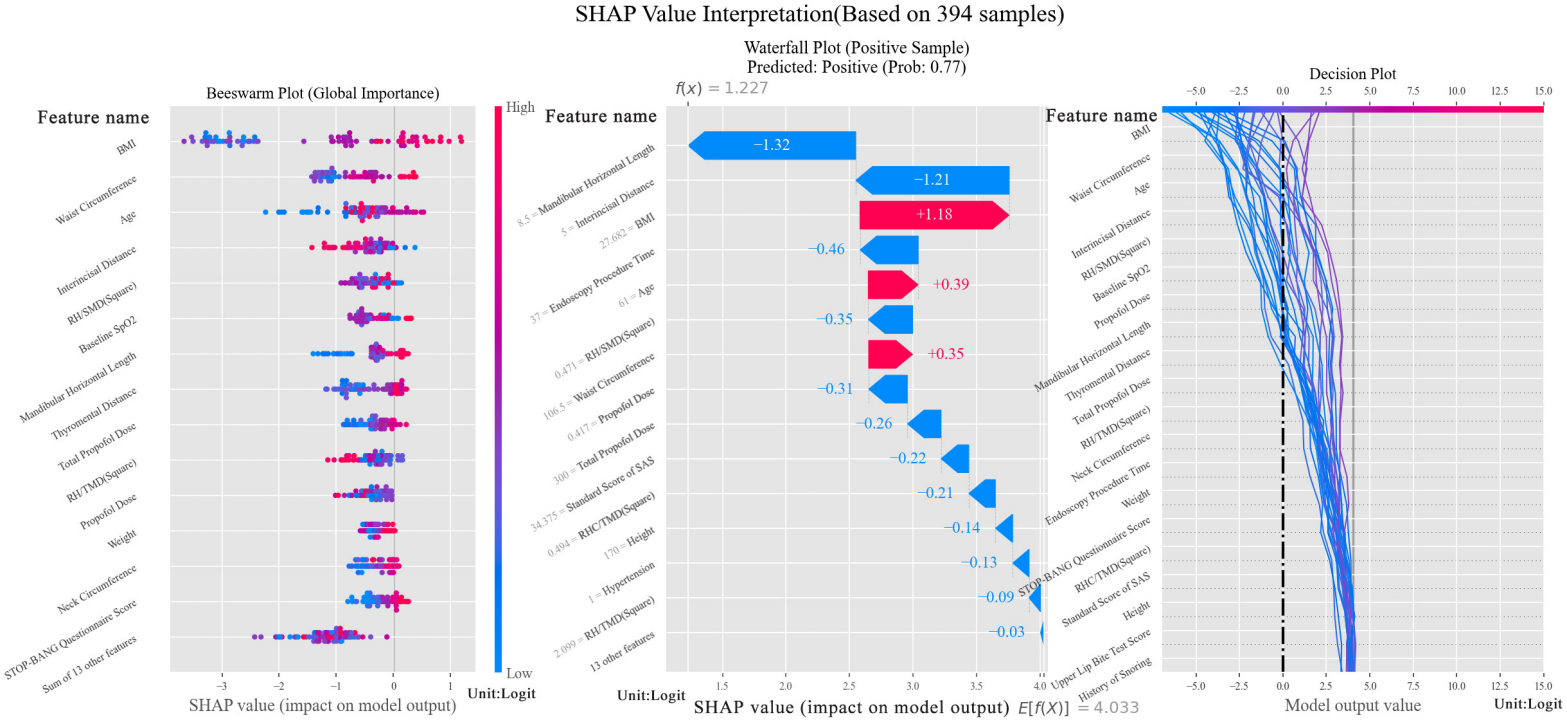
SHAP Value interpretation figure (Left figure is beeswarm, plotted to verify the importance of selected features. The middle figure is a waterfall, which shows the contribution of features to the positive sample. The right figure is a decision figure showing the process of decision of the tree model).

The beeswarm in Figure 2 showcases that selected features hold an effective recognition ability and the waterfall indicates a significant boost for positive classification brought by BMI, age and waist circumference. From decision figure, the decision process is shown directly. Notably, RH/SMD^2^, as an innovative combined feature, exhibits well performance in the feature contribution analysis, ranking the fifth among all the features. Meanwhile, this feature assists the tree model in decision making, which is the sixth important feature among all. Compared with the features proven to be essential in the prediction, RH/SMD^2^ can be used to make a slight adjustment to the predicted probability.

Through feature selection, another notable finding emerged: neck circumference is crucial for predicting hypoxemia. While previous studies have repeatedly emphasized the importance of BMI and waist circumference, neck circumference has not typically been ranked as highly, despite its close association with breathing. Interestingly, its scores surpassed those of age, TMD, and habitual snoring history, as shown in Table 2. Neck circumference ranked in the top three for weight and coverage and ranked fourth for Pearson's Coefficient. Analysis of the three tests revealed that their mean values showed significant differences between positive and negative groups. In the variance test, where most features showed generally high *p*-values, neck circumference had a relatively low *p*-value, further confirming its importance.

**Table 2:** Indicator Score of features proven to be more important.

**Feature level**	**Features**	**Accuracy**	**Precision**	**F1-score**	**ROC–AUC**
Very important (sample = 394)	Age	0.91	0.87	0.82	0.53
	Waist circumference	0.90	0.87	0.88	0.82
	BMI	0.92	0.90	0.90	0.81
	Baseline SpO_2_	0.91	0.82	0.86	0.62
	TMD	0.91	0.82	0.86	0.64
	Habitual snoring history	0.91	0.82	0.86	0.63
Combination	All (sample = 394)	0.93	0.92	0.92	0.85
	All (sample = 647)	0.89	0.88	0.87	0.74
Important (sample = 394)	OSAHS	0.91	0.82	0.86	0.50
	Smoking history	0.91	0.82	0.86	0.55
	Propofol dose	0.91	0.82	0.86	0.66
	Neck circumference	0.91	0.82	0.86	0.73
Innovative airway features (sample = 394)	RH/TMD^2^	0.91	0.82	0.86	0.63
	RHC/TMD^2^	0.91	0.82	0.86	0.58
Selected (sample = 394)	Age	0.89	0.87	0.87	0.74
	Height				
	BMI				
	Propofol induction dose				
	RHC/TMD^2^				
	RH/TMD^2^				
	OSAHS				
	Weight				
	Baseline SpO_2_				
	Waist circumference				
Selected (sample = 647)	Selected features	0.92	0.91	0.90	0.80

To validate the importance of neck circumference, an ablation study was conducted using the selected model. The results are presented after model generalization.

### 4.2 Verification of feature selection

By putting the features in to the XGBoost, the comparative results have been obtained, which are presented in Table 3. A performance comparison between the full feature set and the 29 selected features showed that the selected features contributed more strongly to prediction, reinforcing the reliability of the model.

**Table 3:** The performance contrasts of selected features, all features, as well as some combinations proven to be important (sample = 394).

**Combinations**	**Accuracy**	**Recall**	**F1-score**	**ROC–AUC**
Very important factors	0.89	0.87	0.89	0.65
Important factors	0.86	0.86	0.81	0.49
All features	0.88	0.88	0.86	0.64
Selected features	0.91	0.91	0.91	0.74

The results in Table 3 indicate that the selected features outperformed previously studied risk factors, achieving an accuracy, recall, and F1-score, as well as an AUC of 0.74. However, without CV, these results do not fully represent the generalizability of the model. Despite this limitation, the selected features are still considered robust, as they achieved higher scores across all metrics compared with other features under the same conditions.

Next, the result of the combined features is analyzed:

- The results for features with varying levels of importance are shown in Table 2. As indicated, the “Very Important Risk Factors” yielded higher scores, reaching 0.80 for ROC–AUC, while the scores for “Important Factors” were lower. This finding supports the validity of the proposed model. Notably, neck circumference significantly contributed to hypoxemia prediction. It achieved an accuracy above 0.90, with precision and F1-score both exceeding 0.80, which is comparable to the performance obtained from the combination of “Very Important” risk factors.
- The results of the innovative airway features are also presented in Table 2. As shown, the model using RH/TMD^2^ achieved an ROC–AUC of 0.63, while the other evaluation metrics approached 0.90, comparable to the performance of the “Important Risk Factors” combination. This demonstrates that these innovative airway features exhibit relatively significant potential for predicting hypoxemia.
- The results of various feature combinations are listed in Table 2. Certain combinations achieved higher scores than any single feature, thereby further improving the model's performance.

From Table 2, it can be observed that the accuracy, precision, and F1-score of the model based on the selected features approached 0.90, whereas the ROC–AUC reached 0.74. To account for dataset imbalance, the model was adapted to a new dataset with more positive samples, increasing the total sample size to 647 participants. In this dataset, all evaluation metrics showed slight improvements. Furthermore, the selected features emphasized the significant contribution of multiple feature combinations in maintaining the stability and generalizability of the model.

### 4.3 Model comparison

To verify the benefit of XGBoost, the other two models have been compared, whose hyper-parameters have been tuned to the best. The results based on five evaluation indicators are presented in Table 4. Given the clinical importance of accuracy, recall, and F1-score, XGBoost exhibits the best performance among the three models, which is more suitable for constructing the hypoxemia prediction model.

**Table 4:** The comparison of three models based on all the selected features (sample = 394).

**Models**	**Accuracy**	**Precision**	**Recall**	**F1-score**	**ROC–AUC**
RF	0.89	0.86	0.89	0.87	0.69
SPE	0.83	0.90	0.62	0.86	0.80
XGBoost	0.91	0.87	0.90	0.88	0.81

### 4.4 Model generalization

**Figure 3:**
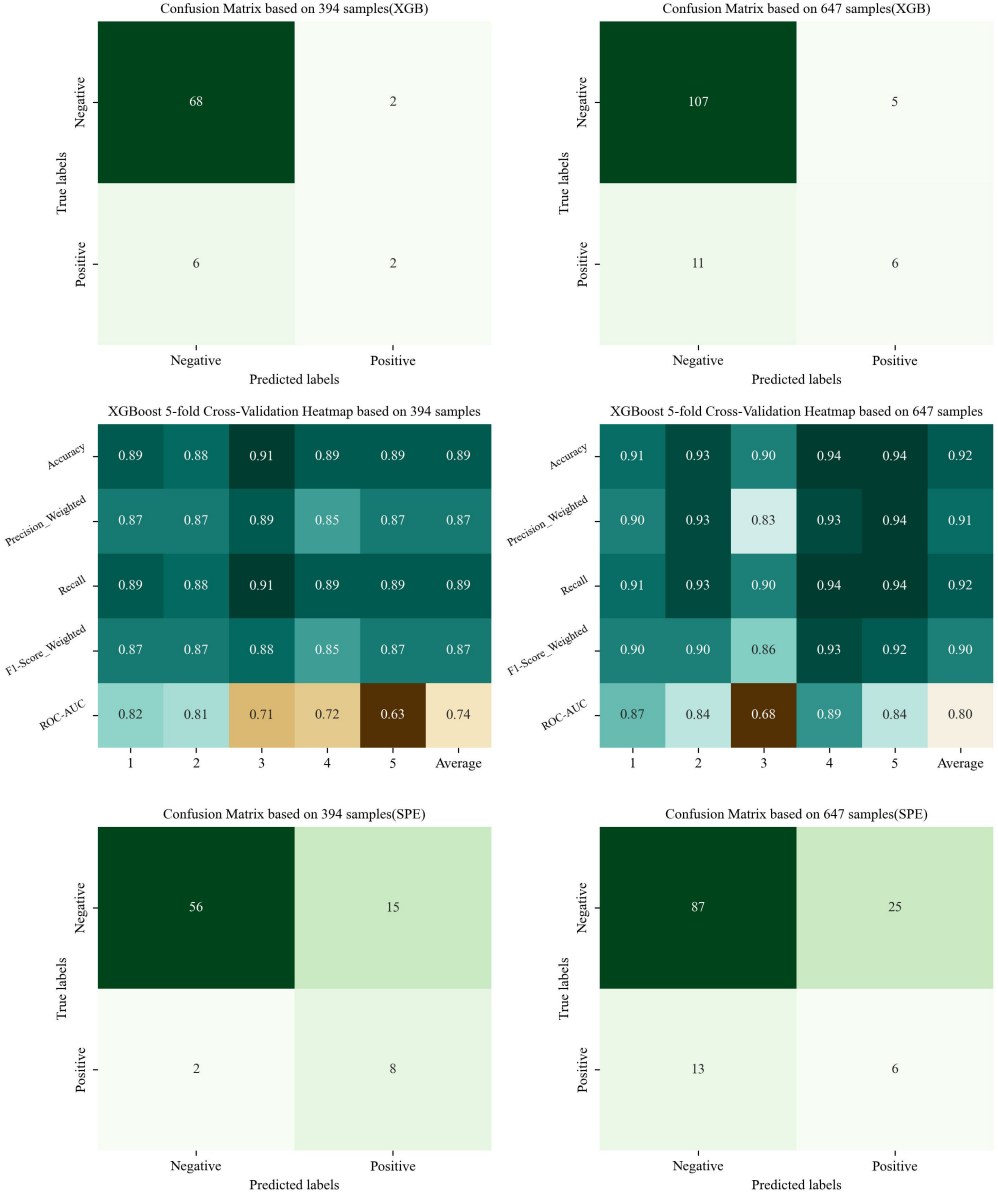
The confusion matrix and indicator heatmap based on XGBoost and SPE. (This figure shows the result of Sections 4.4 and 6: the figures in the first line are the result of Model Generalization, showing the confusion matrix based on XGBoost; the figures in the middle row are the result of model generalization, showing the indicator heatmap of CV; the figures in the last line are the result of Section 6, showing the confusion matrix based on SPE. Figures in the left column utilize the imbalanced dataset of 394 samples, while those in the right column utilize the dataset of 647 samples).

In the preliminary phase, the confusion matrix in Figure 3 was generated using 394 samples and revealed a high FNR. This was largely due to the model's poor performance with small sample sizes of positive samples. To mitigate this issue, additional positive samples were collected, and certain atypical negative samples were removed to reduce class imbalance. The result is shown in Figure 3, which improved the model's accuracy for positive predictions.

The results of five-fold CV are also shown in Figure 3. In these results, the ROC–AUC was slightly low, reflecting the limited ability of the XGBoost model to handle imbalanced datasets. However, when the trained model was adapted to a more balanced dataset containing 647 samples, performance improved. These findings demonstrate that XGBoost can be effective for predicting hypoxemia in clinical settings. Future studies will focus on further improving predictive accuracy by incorporating larger datasets.

**Figure 4:**
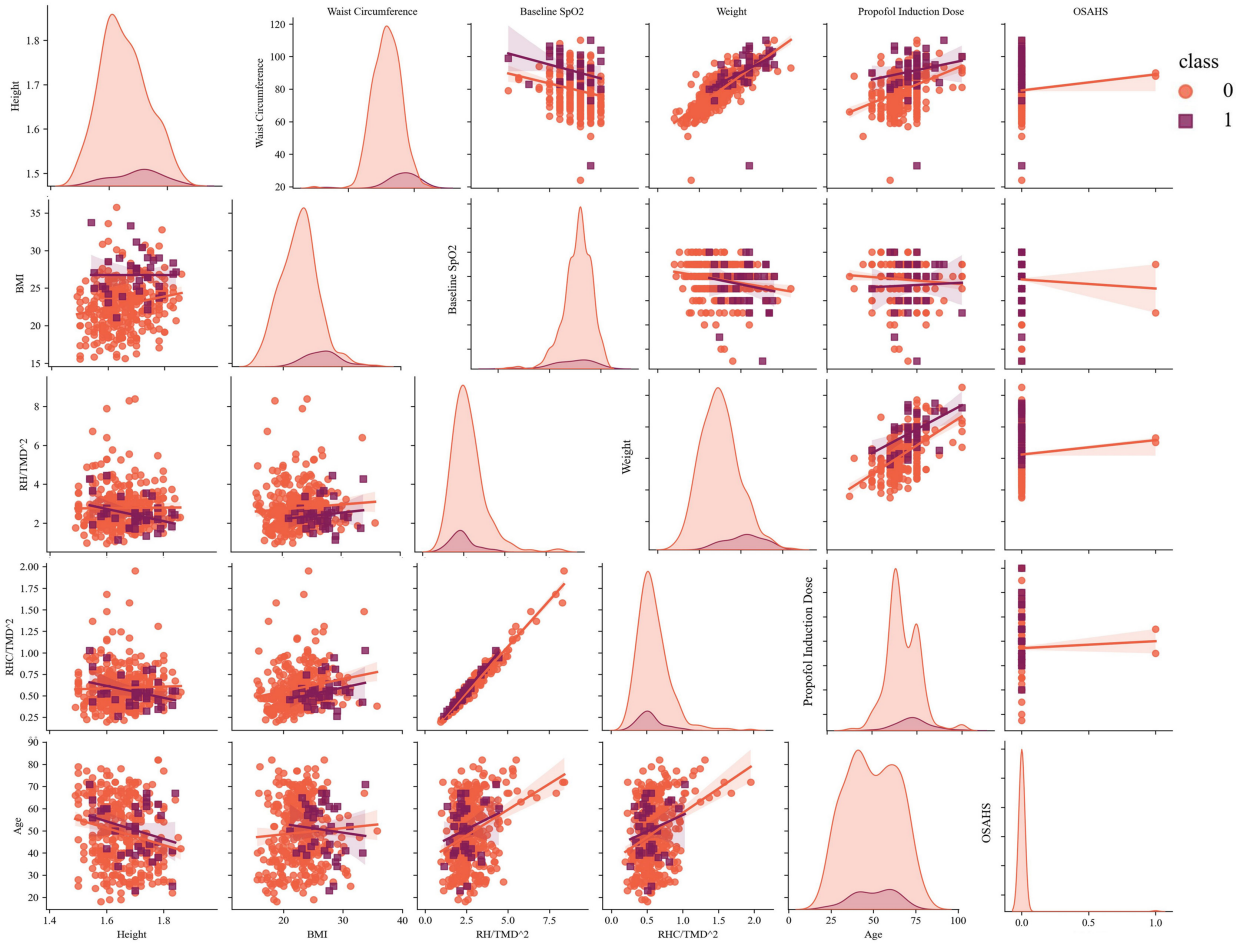
The scatter matrix of selected features and analysis of the fitted intervals (sample = 394).

Additionally, we plotted the scatter matrix of the selected features to analyze the low ROC–AUC from a mathematical perspective, as shown in Figure 4. The scatter distribution indicated that the abnormal samples tended to cluster around the perimeter without forming strong fitting relationships. A wider range of fitted intervals contributed to the low ROC–AUC. Increasing the sample size can help reduce the impact of imbalanced distributions and improve model performance, a finding also supported by the confusion matrix in Figure 3.

From the Table 2, BMI is the most important feature in view of its best score in terms of Accuracy, Precision and F1-score, while waist circumference ranks second according to its best performance in ROC-AUC. To validate this conclusion, an ablation study was conducted using XGBoost with a total of 647 samples. Model performance was assessed using five indicators, and a five-fold CV was applied to evaluate robustness, whose results were as follows. BMI: Accuracy = 0.87, Precision = 0.80, Recall = 0.86, F1-score = 0.83, ROC–AUC = 0.70; waist circumference: Accuracy = 0.85, Precision = 0.81, Recall = 0.83, F1-score = 0.82, ROC–AUC = 0.68. In Section 4.1, the critical role of neck circumference was also emphasized, whose importance is always neglected in the previous studies. To prove this innovative finding, the same method is applied in the neck circumference. The results were as follows: Accuracy = 0.89, Precision = 0.82, Recall = 0.86, F1-score = 0.84, ROC–AUC = 0.73. The results obtained without neck circumference were approximately the same as those without BMI, supporting the previous conclusion that neck circumference is highly important for predicting hypoxemia.

## 5 Discussion

Among the included 647 patients, 72 experienced a drop in SpO_2_ below 95% (11.13%), 43 experienced a drop below 90% (6.65%). No serious sedative-related adverse events occurred in all patients of this study. This study employed an XGBoost-based model to predict hypoxemia during sedated gastrointestinal endoscopy. The key highlights of the study are as follows: First, it was conducted prospectively, which helped avoid potential bias. Second, the XGBoost model demonstrated strong performance in predicting hypoxemia during sedated gastrointestinal endoscopy. Third, BMI, waist circumference, neck circumference, age, baseline SpO_2_ contribute most significantly to the hypoxemia prediction. Among them, BMI and waist circumference rank respectively the first and the second, while neck circumference is often neglected in the previous studies. Fourth, we explored combinations of multiple features across the full feature set and incorporated innovative airway features to enhance prediction, such as RH/SMD^2^ and RH/TMD^2^.

More than 14 million gastrointestinal endoscopies are performed annually in China, and this number is projected to increase to 51 million by 2030 because of the aging population (25). Sedation is administered in more than 48.3% of gastrointestinal endoscopy procedures in China (47.9% for gastroscopy and 49.3% for colonoscopy), compared with 98% in the United States (25–27). Sedated endoscopy not only reduces patient anxiety and fear but also significantly improves comfort and compliance (28, 29). Hypoxemia is the most common complication and can result in serious consequences. Although the clinical use of pulse oximeters enables continuous monitoring of oxygen saturation, hypoxemia remains inadequately predicted (30). Real-time monitoring of blood oxygen saturation with pulse oximeters helps clinicians take immediate measures to shorten hypoxemic episodes (31). However, if hypoxemia can be effectively predicted before onset, clinicians would be able to take preventive measures in advance, thereby reducing patient harm. Thus, clinically predicting and preventing hypoxemia remains challenging, and further efforts are needed to mitigate its occurrence and avoid severe or life-threatening outcomes (32).

Perioperative risk assessment is critical in medical informatics. Previous research has predominantly focused on analyzing the risk factors and developing logistic regression models to predict adverse outcomes associated with anesthesia and sedation during gastrointestinal endoscopy (33). Some studies have used logistic regression to identify three independent risk factors with strong discriminatory power and to develop risk predictors for hypoxemia during sedated gastroscopy, enabling the assessment and forecasting of individual hypoxemia risk in this procedure (16).

Compared with logistic regression analysis, ML techniques provide several advantages, including the ability to handle diverse data types and deliver superior predictive performance (34). ML has been successfully applied in clinical medicine to predict adverse events, such as acute kidney injury and adverse drug reactions (35). One study employed the XGBoost model to identify four easily accessible factors for predicting and screening high-risk outpatients with hypoxemia during sedated colonoscopy (18). Our research expands on this study by focusing on patients who underwent sedated gastrointestinal endoscopy. Risk prediction primarily relies on fundamental patient data (e.g., age and weight), comorbidities (e.g., hypertension, diabetes, and coronary heart disease), and anesthetic agents (e.g., propofol and fentanyl) prior to diagnosis and treatment (6). Despite these advancements, there remains a considerable gap in developing robust ML models for hypoxemia risk prediction. Addressing this gap could yield valuable tools to support clinical decision-making and improve patient outcomes. In this study, we compared three ML models: RF, SPE, and XGBoost. RF, a classical tree-based model, and SPE, a model tackling the imbalance, was set as the control groups. XGBoost, a state-of-the-art tree-based model, was used to evaluate tree model performance without explicitly handling imbalance.

Although prior studies on the clinical applications of ML have shown its potential to generate accurate predictions, the lack of interpretability remains a major barrier to its broad adoption in clinical practice. Explainable algorithms are essential, as they clarify the reasoning behind diagnostic predictions for individual patients and highlight the patient-specific features contributing to outcomes. Without interpretability, advanced methodologies such as deep learning and ensemble models have limited utility in medical decision-support systems.

In our study, we developed a robust hypoxemia prediction model based on XGBoost, given its strong interpretability and outstanding classification capacity, as shown in previous studies. SHAP values, a state-of-the-art tool for interpreting tree models, provided a novel perspective on evaluating the efficiency of the XGBoost-based model. To optimize performance, several hyperparameters—including n-estimators, max-depth, minimum weight of leaves, learning rate (α), γ, λ—were adjusted. After optimization, XGBoost was applied to each selected feature and its combinations to assess feature contributions. Although certain features and combinations demonstrated high outcome accuracy (0.93) or F1-score (0.92), they did not account for ROC–AUC and Recall. Ultimately, the ROC–AUC of our model reached 0.81, while the recall achieved 0.90, which is relatively high for a tree model applied to an imbalanced dataset. Another advantage of our study was that the model combined the relevant indicators of airway assessment, including TMD, mandibular horizontal length, upper lip bite test score, etc (36). we also explored a combination of multiple specific features, such as RH/SMD^2^ and RH/TMD^2^. Prediction tools can be designed to incorporate these variables to improve predictive performance.

Effectively conveying the rationale behind predictions to clinicians is critical. While numerical risk scores are useful, detailed explanations showing how clinical indicators such as BMI, contribute to predictions are of greater clinical value. Such insights are not only more comprehensible and acceptable to medical professionals but also actionable, as some factors can be modified through interventions to reduce risk. Our findings confirmed that BMI, waist circumference, neck circumference, age, baseline SpO_2_ contribute most significantly to the hypoxemia prediction. Among them, BMI is the most important feature, while neck circumference is often neglected in the previous studies. Patients with an elevated BMI are more susceptible to airway obstruction during anesthetic sedation, thereby elevating the incidence of sedation-related adverse events and airway management interventions (37). In the present study, we also found that obese patients require heightened clinical vigilance during sedated gastrointestinal endoscopy. As a body fat index, neck circumference is easy to measure, stable, and less influenced by fasting or respiratory movements. Molnár et al. (38) reported that neck circumference is essential for predicting velopharyngeal obstruction and OSAHS, while Fu et al. (39) demonstrated that it's a better body fat indicator than BMI, particularly for patients undergoing gastrointestinal endoscopy, where sedatives may increase hypoxia risk. Furthermore, the present study demonstrates that waist circumference, serving not only as a key anthropometric indicator of abdominal obesity but also as a significant predictor of hypoxemia during sedated gastrointestinal endoscopy, despite limited prior relevant literature. In clinical practice, waist circumference can serve as a straightforward indicator for risk assessment, particularly applicable to abdominal obese patients with a normal BMI but an elevated waist circumference. Numerous studies have demonstrated that sedation-related adverse events during endoscopic examinations are significantly associated with patient age. This may be attributed to the age-related elevation in the incidence of cardiopulmonary complications (40). Patients with low baseline SpO_2_, who may have comorbid lung diseases or inadequate oxygen reserve, exhibit an elevated risk of hypoxia during sedated endoscopy.

## 6 Limitations

First, it was a single-center study. Previous research has proposed a federated learning algorithm (41) designed to integrate multiple servers to optimize the global model.

Second, we did not perform external validation. Although CV was used to assess generalizability, the performance of this model in external datasets remains uncertain.

Third, owing to the rarity of hypoxemia and the difficulty of collecting positive samples in clinical settings, combined with the limited performance of XGBoost on imbalanced datasets, the model has not yet reached its full potential. To address this limitation, SPE, which is better suited for imbalanced datasets, was applied to the selected features. As shown in Figure 3, this approach improved the prediction of positive samples, although the accuracy of negative samples decreased slightly. In clinical practice, however, prioritizing the identification of patients at risk of complications—even at the cost of a minor reduction in negative prediction accuracy—is a reasonable trade-off for ensuring patient safety. Therefore, future studies will further explore the SPE and latent space analysis. Beside of SPE, other modern imbalance-mitigation strategies, including cluster-based undersampling and Synthetic Minority Over-sampling Technique (SMOTE), have been also tried, but with the poor average performance (Recall = 0.67; F1-score = 0.72). This anomaly can be attributed to the insufficient number of positive samples and the excessive feature-space dimensionality, which jointly inflate the distributional discrepancy. However, in our new studies, latent space based interpolation method has been tried to tackle this issue, exhibiting well potential. In the future work, more details around this upsampling strategie will be further validated so as to be adapted to clinical practice.

Fourth, the inclusion of a large number of features may reduce efficiency. To enhance efficiency, Feature Binding (42), as proposed by LightGBM, is a promising method that warrants testing in future research.

## 7 Conclusion

In this study, we compared three mainstream ML models to predict hypoxemia during gastrointestinal endoscopy under sedation, among which XGBoost exhibits the best performance. Using clinical data from nearly 650 cases collected between January and May 2025 at Shanghai Sixth People's Hospital, affiliated with Shanghai Jiao Tong University School of Medicine, we identified 29 key features through statistical analysis and ML analysis. In view of the strong performance and generalizability for clinical applications, XGBoost was selected as the prediction model. By adapting the model to three levels of features, 10 features were combined to enhance its performance, some of which were innovative airway features not previously used in similar studies. Notably, BMI is the most important feature and waist circumference follows. Meanwhile, neck circumference, which is often ignored in the relative studies, emerged as a new clinically significant factor.

In the future, we plan to further evaluate the SPE, which demonstrated potential in model validation, through additional studies. Latent-space analysis, a novel method for addressing data imbalance, will also be investigated in future studies. Furthermore, refining the XGBoost model with larger and more diverse datasets is expected to improve accuracy, recall, ROC–AUC, and the overall robustness of clinical predictions.

## Data Availability

The original contributions presented in the study are included in the article/supplementary material, further inquiries can be directed to the corresponding authors.
